# Determinants of Quality of Work Life among Nurses in Intensive Care Units

**DOI:** 10.1192/j.eurpsy.2026.10781

**Published:** 2026-07-31

**Authors:** B. K. Jihene, C. Farah, R. Nakhli, B. Narjess, C. Sridi, G. Naoures, F. Amen, I. Kacem, M. Maoua

**Affiliations:** 1 Clinical Psychology Unit, Occupational Medicine Department; 2 Occupational Medicine Department, Sahloul University Hospital; 3 Faculty of Medicine of Sousse, University of Sousse; 4 Laboratoire de Recherche (LR19SP03); 5 Occupational Medicine Department, Farhat Hached University Hospital, Sousse, Tunisia

## Abstract

**Introduction:**

Occupational stress represents a major challenge in workplace health, particularly within hospital settings where nurses are highly exposed. Coping strategies play a pivotal role in adapting to stressful professional demands and in preventing burnout.

**Objectives:**

This study aimed to describe coping strategies used in stress management and in the prevention of professional burnout among nurses.

**Methods:**

We conducted a quantitative, descriptive, cross-sectional, and analytical survey using a self-administered questionnaire incorporating the Ways of Coping Checklist (WCC) developed by Lazarus and Folkman. This tool assesses problem-focused and emotion-focused coping mechanisms in stressful situations. The study included 100 nurses working in various departments of Sahloul University Hospital during November and December 2024. Data were analyzed using SPSS software (version 25).

**Results:**

The study population was predominantly female (69%, sex ratio = 0.44). Seventy two percent of participants (72%) were aged 30–39 years, with a mean age of 35.2 years. Almost half of the nurses described their work as “tiring” (49%) or “exhausting” (47%). The main stressors identified were high workload (57%) and emotional burden (29%). Only 10% of nurses had received specific training in stress management. Based on the applied criteria, 88% of participants presented with burnout syndrome. Coping assessment revealed that problem-focused coping was the preferred strategy for 61% of respondents.

**Conclusions:**

These findings highlight the need to implement targeted training programs focused on managing negative emotions and stress adaptation techniques. Strengthening the use of problem-focused coping could improve emotional regulation, reduce the risk of burnout, and ultimately enhance nurses’ quality of work life

**Disclosure of Interest:**

None Declared

